# Surgical Excision of Orbital Progressive Granular Cell Tumour

**DOI:** 10.1155/2015/420490

**Published:** 2015-05-18

**Authors:** Demetrio Germanò, Hossein Mostafa Elbadawy, Diego Ponzin, Daniele Ferro, Leonardo Priore

**Affiliations:** ^1^The Department of Maxillofacial Surgery, Ospedale dell'Angelo, Via Paccagnella 11, 30174 Venice, Italy; ^2^The Veneto Eye Bank Foundation, Via Paccagnella 11, 30174 Venice, Italy

## Abstract

Granular cell tumour (GCT) is mostly benign lesion first described by Abrikossoff and named after him. Most cases are reported in the head and neck area, where the tongue is the most common site. Here we review previous cases in the literature for GCT in the orbit and present a new case. A 49-year-old male presented with apparent exophthalmos. Examination of the patient revealed the presence of a mass in the bottom side of the orbit. A substantial progress was noted after two months from the initial examination using computed tomography (CT) scan. An orbital mass was extracted and histological analysis showed signs typical for GCT. Immunohistochemistry was positive for S-100; the biopsy showed no mitotic or necrotic areas. Proptosis was resolved after surgery and a six-year follow-up CT scan was performed. We conclude that rapid progress of the tumour does not necessarily suggest malignancy.

## 1. Introduction

Granular cell tumour (GCT) is a commonly benign soft tissue lesion of neural origin according to histochemical and ultrastructure studies [1]. It was first described by Abrikossoff [2] as a granular cell myoblastoma. Up to 10% of granular cell tumours are multiple [3–5]. A slight female predominance exists, with an estimated female-to-male ratio of approximately 3 : 2. Granular cell tumours affect persons of a wide range of ages. Most patients are middle-aged, with a peak incidence in the fourth through the sixth decades of life. It is widely accepted that the GCT can occur virtually in all parts of the body; nevertheless, head and neck areas are the most affected sites by this type of tumours, with more than two-thirds of those cases diagnosed in the tongue [6]. GCT in the orbit is very rare [7–9]; additionally, malignant orbital GCT was scarcely reported [10–12]. Histological specimen examination and the rate of tumour progress can be two indicative parameters for determining the malignancy of a tumour as early as possible to avoid metastases [11]. Slowly developing tumour can be suggestive of classification as a benign like the majority of GCT. Here we describe the excision of an orbital GCT which rapidly progressed within two months from the first diagnosis. We also report the follow-up of the case for 6 years after surgery.

## 2. Case Presentation 

**Figure 1:**
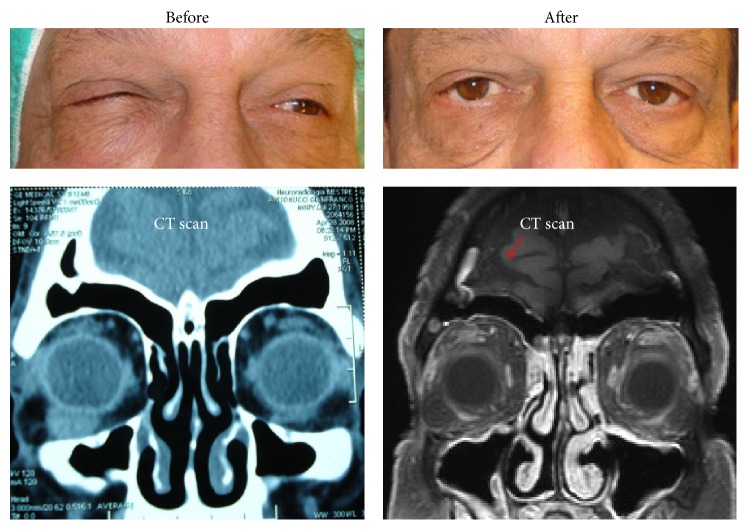
GCT before and after surgery. Top panel shows proptosis before (left) and after (right) surgery. Bottom panel shows CT scans to identify the GCT (left), and to confirm the absence of the mass after surgery (right).

**Figure 2:**
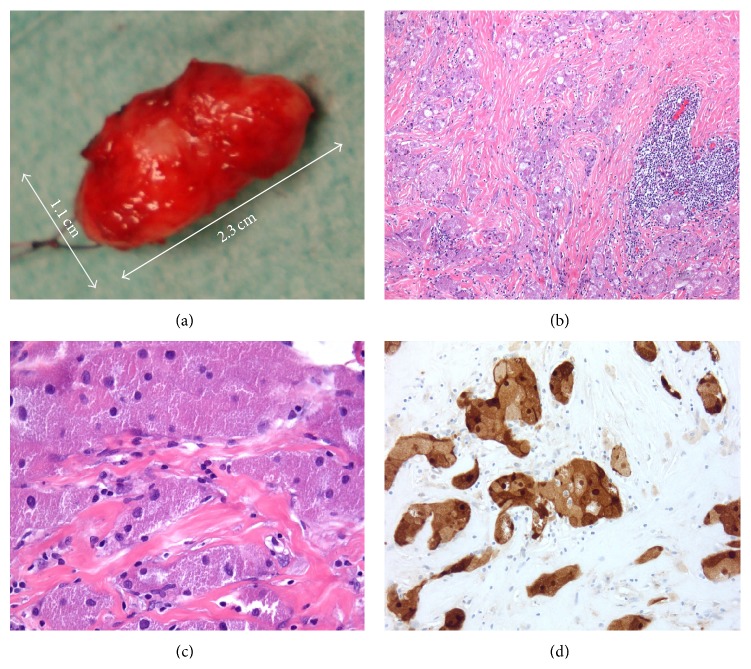
Histological analysis of the excised mass. Macroscopic examination, showing size and morphology of the mass (a). Histological analysis showed typical GCT histology with granular cytoplasm. Cells were imbedded in a connective tissue bed (b) with batches of high density of nuclei. Cells were large in size with small nuclei and granular cytoplasm (c). Immunohistochemical staining against S-100 protein was positive in the cytoplasm (d).

A 49-year-old male patient presented with a history of progressive exophthalmos. Initially, he presented with a minor proptosis. Two months later, a significant progress was noted and ophthalmologic examination revealed a subcutaneous mass with partial occlusion of the lids (Figure 1). Vision acuity was 8/10; however, full ophthalmic examination was not performed due to proptosis and diplopia. The first computed tomography (CT) scan revealed the presence of a spherical solid tumour close to the inferior temporal orbit, with 10.40 mm in length and 7.99 mm diameter. The mass (Figure 2) was extracted immediately and histological analysis was carried out. Upon surgical excision of the mass from the bottom of the eye globe the differential diagnosis was suggestive of subcutaneous cysts, granuloma, schwannoma, fibrous histiocytoma, or neurofibroma. Histological staining of the specimen showed that the cells had granular cytoplasm with ill-defined borders, typical for GCT. Immunohistochemistry showed S-100 positive staining (Figure 2). The excisional biopsy showed no mitosis or necrotic areas. Proptosis was resolved after surgery and CT scan showed no signs for the presence of GCT (Figure 1). After one year, the vision was restored to 10/10 and there was no sign of diplopia. Regular follow-up for six years following the surgery showed no signs of recurrence of the tumour, which further confirmed the benign nature of the extracted mass.

## 3. Discussion

Abrikossoff's tumour is a rare tumour with commonly benign evolution. CT scan is essential for the correct localization of the mass and early diagnosis is very important to preserve the eye globe. The aim of this report is to describe a case of granular cell tumour in the orbit, to provide a comprehensive summary of previous reports of this case, and to review the malignancy incidence rate of this tumour. The case presented here was suspected to be a malignant tumour due to its fast progress within two months from the first examination; however, histological analysis suggested that it was a benign tumour. This was confirmed by examining the patient regularly for six years. Benign and malignant counterparts are known; the latter are rare, comprising fewer than 2% of all granular cell tumours. In fact, according to previously reported data (Table 1), 3 out of 31 cases were malignant GCT (9.68%) in the past 20 years. Therefore, malignancy cannot be ruled out of the differential diagnosis.

Literature review of previous 31 cases shows no age related incidence of GCT in the orbit. Cases reported in age range from 4 years to 72 years, with most cases being reported in patients with 40 years or more. Three cases of GCT malignancy were reported, and the latest was in the year 2000 (Table 1).

## Figures and Tables

**Table 1:** 

Year	Number of cases	Age	Malignancy	Reference
1975	1	8	Benign	[13]
1987	1	44	Benign	[14]
1987	6	—	Benign	[15]
1983	1	65	Benign	[7]
1983	1	42	Benign	[16]
1991	4	—	Benign	[8]
1991	4	>4	Benign	[17]
1994	1	Unknown	Malignant	[11]
1996	1	30	Malignant	[12]
1997	1	43	Benign	[18]
2000	1	72	Malignant	[10]
2004	1	67	Benign	[19]
2005	1	56	Benign	[20]
2006	1	49	Benign	[21]
2007	1	26	Benign	[22]
2011	1	65	Benign	[23]
2012	1	53	Benign	[24]
2012	1	Unknown	Benign	[25]
2013	1	50	Benign	[26]
2013	1	42	Benign	[27]
